# Physical frailty and cognitive impairment in older nursing home residents: a latent class analysis

**DOI:** 10.1186/s12877-021-02433-1

**Published:** 2021-09-07

**Authors:** Yiyang Yuan, Kate L. Lapane, Jennifer Tjia, Jonggyu Baek, Shao-Hsien Liu, Christine M. Ulbricht

**Affiliations:** 1 Clinical and Population Health Research PhD Program, Graduate School of Biomedical Sciences, University of Massachusetts Medical School, Worcester, MA USA; 2 Department of Population and Quantitative Health Sciences, University of Massachusetts Medical School, 368 Plantation Street, Worcester, MA 01605 USA; 3 National Institute of Mental Health, National Institutes of Health, Bethesda, MD USA

**Keywords:** Physical frailty, Cognitive impairment, Latent class analysis, Nursing home

## Abstract

**Background:**

Little is known about the heterogeneous clinical profile of physical frailty and its association with cognitive impairment in older U.S. nursing home (NH) residents.

**Methods:**

Minimum Data Set 3.0 at admission was used to identify older adults newly-admitted to nursing homes with life expectancy ≥6 months and length of stay ≥100 days (*n* = 871,801). Latent class analysis was used to identify physical frailty subgroups, using FRAIL-NH items as indicators. The association between the identified physical frailty subgroups and cognitive impairment (measured by Brief Interview for Mental Status/Cognitive Performance Scale: none/mild; moderate; severe), adjusting for demographic and clinical characteristics, was estimated by multinomial logistic regression and presented in adjusted odds ratios (aOR) and 95% confidence intervals (CIs).

**Results:**

In older nursing home residents at admission, three physical frailty subgroups were identified: “mild physical frailty” (prevalence: 7.6%), “moderate physical frailty” (44.5%) and “severe physical frailty” (47.9%). Those in “moderate physical frailty” or “severe physical frailty” had high probabilities of needing assistance in transferring between locations and inability to walk in a room. Residents in “severe physical frailty” also had greater probability of bowel incontinence. Compared to those with none/mild cognitive impairment, older residents with moderate or severe impairment had slightly higher odds of belonging to “moderate physical frailty” [aOR (95%CI)_moderate cognitive impairment_: 1.01 (0.99–1.03); aOR (95%CI)_severe cognitive impairment_: 1.03 (1.01–1.05)] and much higher odds to the “severe physical frailty” subgroup [aOR (95%CI)_moderate cognitive impairment_: 2.41 (2.35–2.47); aOR (95%CI)_severe cognitive impairment_: 5.74 (5.58–5.90)].

**Conclusions:**

Findings indicate the heterogeneous presentations of physical frailty in older nursing home residents and additional evidence on the interrelationship between physical frailty and cognitive impairment.

**Supplementary Information:**

The online version contains supplementary material available at 10.1186/s12877-021-02433-1.

## Introduction

Over 1.2 million U.S. older adults aged ≥65 years reside in a nursing home (NH) [1]. Physical frailty, characterized by decreased physiologic reserve and increased vulnerability to exogenous stressors [2], and cognitive impairment, ranging from mild cognitive impairment to fully-developed dementia [3], are the two most prominent conditions in this population. Both are highly prevalent, with 30–85% of older nursing home residents experiencing physical frailty [4–6] and 65% moderate to severe cognitive impairment [1]. Both are associated with adverse health outcomes, including lowered quality of life and elevated risks for hospitalization and mortality [4, 7–9].

Physical frailty may encompass weakness, slowness, low level of physical activity, weight loss and exhaustion [2], and older adults may experience the heterogeneous symptoms [10, 11]. Latent class analysis (LCA) can help identify subgroups of older adults with distinct clinical profiles of physical frailty by using observed symptom indicators. For example, in a cohort of community-dwelling older adults in Taiwan, three physical frailty subgroups were identified with LCA: one characterized by slowness and weakness, one weight loss and exhaustion, and one low physical activity [10]. For older U.S. nursing home residents, whether physical frailty has similar heterogeneous clinical presentations remains unknown.

A better understanding of the multifaceted presentations of physical frailty and can inform its management. Studies have demonstrated improvement in physical frailty in community-dwelling older adults with exercised-based interventions [12, 13]. However, interventions shown to be effective for specific physical frailty domains, such as muscle strength, physical activity, gait speed, and energy [14–17], may not be as effective for other domains. Research on the heterogeneous profile of physical frailty in older nursing home residents can be informative for the development of tailored planning of care.

Physical frailty and cognitive impairment share many risk factors, often co-occur, and predict the onset of each other [3, 18–21]. Given this interrelationship between these two conditions, older adults’ physical frailty symptoms may be associated with levels of cognitive impairment. Physical frailty may have distinct clinical manifestations in older residents with different cognitive impairment levels, or physical frailty may have consistent symptom profiles, but older adults with severe cognitive impairment may have higher odds of experiencing a particular profile. However, no studies have quantified this interrelationship. Additionally, the construct “cognitive frailty” has been proposed by the International Academy on Nutrition and Aging (IANA) and the International Association of Gerontology and Geriatrics (IAGG) to capture the co-existence of physical frailty and mild cognitive impairment in the absence of overt dementia and other neurological conditions [22]. However, to date, there is no consensus on the operationalization of “cognitive frailty” [23], leading to discrepancies in the estimates of its prevalence and associations with adverse health outcomes in community-based studies [24]. Treating two conditions as separate constructs with LCA to examine whether and to what extent the heterogeneity of physical frailty is associated with the severity of cognitive impairment could provide insight on the underlying mechanisms behind the observed interrelationship between the two conditions, as well as implications to have the personalized management for specific physical frailty subgroups by the level of cognitive impairment.

This study thus sought to use LCA to explore the heterogeneity of physical frailty and its association with cognitive impairment in older U.S. nursing home residents. The objectives were to identify subgroups of physical frailty, and examine if these subgroups varied by cognitive impairment in newly-admitted, long-stay older nursing home residents.

## Methods

The University of Massachusetts Medical School Institutional Review Board approved this study as exempt from Federal regulations (09/20/2019).

### Data

Minimum Data Set (MDS) 3.0 is mandated for all Medicaid/Medicare-certified U.S. nursing homes. It is conducted at admission and periodically during the nursing home stay, collecting data on residents’ demographic and clinical characteristics, including physical functioning, cognitive functioning, bladder and bowel conditions, nutritional status, pain, diagnoses, and receipt of medications [25].

### Sample

We first identified residents who were “newly-admitted” during 01/01/2014 to 12/31/2016 and aged ≥65 years at admission. “Newly-admitted” was defined as no nursing home stays in ≥90 days prior to the given admission. We excluded those who stayed in the nursing homes for ≤100 days to focus on the “long-stay” older residents [26], and those with a physician-documented prognosis of life expectancy of less than 6 months at admission (MDS 3.0 Section J), as they may be terminally ill and need special care from hospice or palliative services. Older residents who were comatose were also excluded. If a resident had multiple nursing home stays meeting these criteria, the first one was selected (Supplement Figure S.1). The final sample included 871,801 older residents. Their MDS 3.0 assessment at nursing home admission were used in the analysis.

### Measures

#### Physical frailty

FRAIL-NH uses MDS 3.0 items with comparable performance as other well-established metrics such as the Frailty Phenotype and the Frailty Index in assessing physical frailty in nursing home residents [4, 27–33]. Each item (*F*atigue, *R*esistance, *A*mbulation, *I*ncontinence, *L*oss of weight, *N*utritional approach and *H*elp with dressing) was individually scored [34] (Supplement Table S.1). To describe the prevalence of physical frailty, the individual item scores were summed (score range: 0–13) and categorized as robust (0–5), pre-frail [6, 7] and frail (≥8) [4]. In fitting LCA models to identify physical frailty subgroups, each item was used as an observed indicator.

#### Cognitive impairment

MDS 3.0 contains two validated instruments for cognitive impairment: Brief Interview for Mental Status (BIMS; score range: 0–15), administered when residents can self-report their cognitive status [35], and Cognitive Performance Scale (CPS; score range: 0–6), completed by staff when residents cannot participate in BIMS [36, 37]. BIMS and CPS highly correlate with the widely-used clinical tools for cognitive function, such as the Mini-Mental State Examination (MMSE) and the Modified Mini-Mental State Examination (3MS) [35–38]. Combining BIMS and CPS, cognitive impairment was measured in three levels in accordance with the Centers for Medicare & Medicaid Services Nursing Home Data Compendium [1]: none/mild (BIMS 13–15/CPS 0–2), moderate (BIMS 8–12/CPS 3–4), and severe (BIMS 0–7/CPS 5–6) cognitive impairment.

#### Demographic and clinical characteristics

We examined age, sex, race/ethnicity, urban/rural nursing home, admission source, active diagnoses, any presence of pain, and receipt of antipsychotics, antianxiety medications, and antidepressants in past 7 days or since admission. Admission sources included community, acute hospital, or other [including another nursing home/swing bed, psychiatric hospital, inpatient rehabilitation facility, intellectual disabilities and developmental disabilities (ID/DD) facility, long-term care hospitals, or hospice]. Active diagnoses were physician-documented diagnoses deemed relevant to residents’ current health status and care management, including cancer, heart failure, hypertension, diabetes mellitus, Alzheimer’s disease, cerebrovascular accident/transient ischemic attack (TIA)/stroke, non-Alzheimer’s/other dementia (including vascular or multi-infarct dementia, mixed dementia, frontotemporal dementia, Pick’s disease, and dementia related to stroke, Parkinson’s or Creutzfeldt-Jakob diseases), multiple sclerosis, Parkinson’s disease, seizure disorder/epilepsy, arthritis, osteoporosis, hip fracture, other fracture, asthma/chronic obstructive pulmonary disease (COPD)/chronic lung disease, anxiety disorder, and depression.

### Statistical analysis

Analyses were conducted in SAS 9.4 [39] and Mplus 8.4 [40].

#### Main analysis

We described the sample demographic and clinical characteristics at nursing home admission. We then showed the observed frequencies of each FRAIL-NH item for all residents and by cognitive impairment levels.

We used LCA to identify latent subgroups of physical frailty at admission using FRAIL-NH items as the observed indicators. LCA models with 2 to 6 subgroups were fitted and compared to determine the optimal number of physical frailty subgroups. For each model, we obtained (1) fit statistics: entropy, Akaike Information Criterion (AIC), Bayesian Information Criterion (BIC), and sample-size adjusted BIC; (2) subgroup prevalence: the proportion of residents with higher probabilities of belonging to the given subgroup; (3) item-response probability for each indicator by subgroup. After considering model fit, parsimony, and clinical relevance, the best-fitting model was selected, and the optimal number of physical frailty subgroups was identified. We assigned qualitative labels to describe each subgroup based on the overall patterns of the item-response probabilities [41].

We then examined if and how the subgroups would differ by severity of cognitive impairment. First, we fit LCA models within subsets of residents by their cognitive impairment levels; then, in the entire sample, we examined the measurement invariance assumption by evaluating cognitive impairment as a grouping variable (see details in Supplement Method). Because the subgroups of physical frailty did not vary across cognitive impairment levels, we included cognitive impairment as a covariate [41] to assess its association with the identified physical frailty subgroups using multinomial logistic model, adjusting for demographic and clinical characteristics. Results were presented in adjusted odds ratio (aOR) and 95% confidence interval (95%CI).

#### Sensitivity analysis

Consistent with the “cognitive frailty” concept by IANA and IAGG, we conducted three sets of sensitivity analysis by (A) excluding older residents with diagnosis of Alzheimer’s disease (*n* = 767,034); (B) excluding older residents with diagnosis of non- Alzheimer’s/other dementia (*n* = 529,832); (C) excluding older residents with diagnosis of Alzheimer’s disease and those with non- Alzheimer’s/other dementia (*n* = 460,612). In each subsample, LCA models were fit to identify the physical frailty subgroup, and the association between cognitive impairment and the identified subgroups were assessed following the same steps as the main analysis.

## Results

### Sample characteristics

As shown in Table 1, of the 871,801 newly-admitted older residents, 44.3% were ≥ 85 years old, 65.3% women, and 18.8% racial/ethnic minority. Approximately three quarters of residents entered urban nursing homes. Nearly two-thirds were admitted from acute hospitals and less than one in five from the community. At admission, nearly two thirds of residents were physically frail, one in four was pre-frail, and over a third had severe cognitive impairment. About 45% of older residents had more than two physician-documented active diagnoses. Two in five reported presence of pain. Receipt of antidepressants (43.9%), antipsychotics (19.2%) and antianxiety medications (18.4%) were common.

**Table 1:** Demographic and clinical characteristics of newly-admitted older nursing home residents (2014–2016)

	All
	(*n* = 871,801)
	*Percentage*
**Age (years)**	
65 - < 75	21.4
75- < 85	34.3
≥ 85	44.3
**Female**	65.3
**Racial/ethnic minority**	18.8
**Nursing home location**	
Rural	23.5
Urban	76.5
**Admission source**	
Community	17.8
Acute hospital	63.8
Other^a^	18.4
**Physical frailty**	
Robust	9.6
Pre-frail	25.0
Frail	65.4
**Cognitive impairment**	
None/Mild	33.6
Moderate	30.1
Severe	36.4
**Active diagnosis**	
Cancer	6.8
Heart failure	18.9
Hypertension	76.3
Diabetes mellitus	31.3
Alzheimer’s disease	12.0
Cerebrovascular accident/Transient ischemic attack/Stroke	13.9
Non-Alzheimer’s/other dementia^b^	39.2
Multiple sclerosis	0.5
Parkinson’s disease	5.8
Seizure disorder/Epilepsy	5.8
Arthritis	26.1
Osteoporosis	11.7
Hip fracture	5.4
Other fracture	8.2
Asthma/Chronic obstructive pulmonary disease/Chronic lung disease	19.6
Anxiety disorder	21.8
Depression	36.3
**Any presence of pain**	40.9
**In past 7 days or since admission, receipt of …**	
Antipsychotics	19.2
Antianxiety medications	18.4
Antidepressant	43.9

^a^Included another nursing home/swing bed, psychiatric hospital, inpatient rehabilitation facility, ID/DD facility, long-term care hospitals, hospice, and other unspecified admission sources

^b^Included non-Alzheimer’s dementia (e.g. vascular or multi-infarct dementia), mixed dementia; frontotemporal dementia (e.g. Pick’s disease), and dementia related to stroke, Parkinson’s or Creutzfeldt-Jakob diseases

### Indicators of physical frailty

Of all residents, 62.1% did not experience fatigue, 91.5% needed physical assistance to transfer between surfaces, 85.7% could not walk between locations in a room, 57.5% experienced bowel incontinence, 3.0% lost at least 5% of weight in the past 3 months or 10% of weight in the past 6 months, 67.8% were on a regular diet, and 95.2% needed help with dressing. Similar distributions were observed across cognitive impairment level except for a few items. For older adults with severe cognitive impairment, there were higher proportions who did not experience fatigue, had bowel incontinence, and needed mechanically altered diet (Table 2).

**Table 2:** Physical frailty indicators by cognitive impairment in newly-admitted older nursing home residents (2014–2016)

		All	Cognitive impairment		
			None/Mild	Moderate	Severe
		(*n* = 871,801)	(*n* = 292,548)	(*n* = 262,307)	(*n* = 316,946)
		*Percentage*	*Percentage*	*Percentage*	*Percentage*
**FRAIL-NH Items**					
**Fatigue**					
0	No (never or 1 day)	62.1	59.4	59.5	67.1
1	Yes (several days/everyday)	31.7	34.5	33.8	27.2
2	PHQ-9 ≥ 10	6.2	6.1	6.8	5.7
**Resistance**^a^					
0	Independent	4.5	4.5	4.5	4.5
1	With set-up only	4.0	3.9	4.0	4.1
2	Need physical assistance	91.5	91.6	91.5	91.4
**Ambulation**^b^					
0	Independent	6.5	7.9	6.4	5.3
1	With assistive device	7.8	7.3	8.3	7.8
2	Cannot walk	85.7	84.8	85.4	86.8
**Incontinence**					
0	None	19.8	27.3	19.6	13.2
1	Urinary incontinence only	22.7	26.1	23.5	19.0
2	Bowel incontinence	57.5	46.6	57.0	67.9
**Loss of weight**					
0	None	97.0	96.9	97	97.1
1	≥5% past 3 mo./ ≥10% past 6 mo.	3.0	3.2	3.0	2.9
**Nutritional approach**					
0	Regular diet	67.8	76.7	67.6	59.7
1	Mechanically altered diet	26.9	19.4	27.6	33.2
2	Require feeding tube	5.3	3.9	4.8	7.1
**Help with dressing**					
0	Independent	1.8	2.6	1.8	1.1
1	Need help with set up only	3.0	3.5	3.1	2.4
2	Need physical help	95.2	93.9	95.0	96.5

*PHQ-9* Patient Health Questionnaire

^a^Measures if the resident needs assistance to be transferred from one location to another

^b^Measures if the resident can walk in a room

### Subgroups of physical frailty

For model selection, although entropy favored the 2-subgroup model, a clinically relevant subgroup emerged in the 3-subgroup model based on the item response probabilities. While AIC/BIC/adjusted BIC values favored models with more subgroups, for models with 4–6 subgroups, at least two of the identified subgroups largely overlapped and lacked sufficient separation. With these considerations, we chose the 3-subgroup model to represent physical frailty subgroups in nursing home residents at admission (Supplement Table S.2).

Based on the item-response probabilities, we assigned qualitative labels to the three subgroups: “mild physical frailty”, “moderate physical frailty” and “severe physical frailty”. (Table 3) About 7.6% of older residents had higher probabilities to belong to the “mild physical frailty” subgroup, 44.5% to the “moderate physical frailty” subgroup, and 47.9% to the “severe physical frailty” subgroup. The major difference between the “mild physical frailty” subgroup and the other two subgroups were reflected in the probabilities for resistance and ambulation: older adults that were likely to be in the “moderate physical frailty” or the “severe physical frailty” subgroups had high probabilities of needing physical assistance to transfer between locations and inability to walk in a room. The “moderate physical frailty” subgroup and the “severe physical frailty” subgroup were mainly distinguished by the item-response probability for the incontinence item: residents belonging to the “moderate physical frailty” subgroup had about an equal probability of having no urinary incontinence, urinary incontinence only, or urinary and bowel incontinence, while the “severe physical frailty” subgroup had a high probability of both urinary and bowel incontinence.

**Table 3:** Physical frailty 3-class latent class model: subgroup prevalence and item-response probabilities of indicators

		Mild physical frailty subgroup	Moderate physical frailty subgroup	Severe physical frailty subgroup
**Subgroup prevalence**		7.6%	44.5%	47.9%
**Item-response probabilities**				
Fatigue				
0	No (never or 1 day)	**0.74**^a^	**0.61**^a^	**0.62**^a^
1	Yes (several days/everyday)	0.22	0.34	0.31
2	PHQ-9 ≥ 10	0.04	0.06	0.07
Resistance^b^				
0	Independent	**0.56**^a^	0.00	0.00
1	With set-up only	0.33	0.03	0.00
2	Need physical assistance	0.11	**0.96**^a^	**1.00**^a^
Ambulation^c^				
0	Independent	**0.53**^a^	0.05	0.01
1	With assistive device	0.18	0.13	0.01
2	Cannot walk	0.29	**0.82**^a^	**0.98**^a^
Incontinence				
0	None	**0.67**^a^	0.30	0.03
1	Urinary incontinence only	0.22	**0.38**^a^	0.08
2	Bowel incontinence	0.11	0.32	**0.89**^a^
Loss of weight				
0	None	**0.98**^a^	**0.98**^a^	**0.96**^a^
1	≥ 5% past 3 mo./ ≥10% past 6 mo.	0.02	0.02	0.04
Nutritional approach				
0	Regular diet	**0.90**^a^	**0.85**^a^	**0.49**^a^
1	Mechanically altered diet	0.10	0.15	0.41
2	Require feeding tube	0.01	0.01	0.11
Help with dressing				
0	Independent	0.24	0.00	0.00
1	Need help with set up only	0.34	0.01	0.00
2	Need physical help	**0.42**^a^	**0.99**^a^	**1.00**^a^

*PHQ-9* Patient Health Questionnaire

^a^The level of the given indicator with the highest item-response probability. Residents belonging to the given subgroup had the highest probability of experiencing this level of the indicator

^b^Measures if the resident needs assistance to be transferred from one location to another

^c^Measures if the resident can walk in a room

In sensitivity analysis when older residents with Alzheimer’s disease and/or those with non-Alzheimer’s/other dementia were excluded, the three-subgroup model appeared to best fit all three subpopulations (Supplement Table S.3). The overall patterns of the item-response probabilities and the respective prevalence of the physical frailty subgroups were similar and consistent with the full sample: “mild physical frailty” (prevalence range: 6.4–7.2%), “moderate physical frailty” (45.0–47.4%), and “severe physical frailty” (46.1–47.7%) (Supplement Table S.4).

### Association between physical frailty subgroups and cognitive impairment

The three subgroups appeared consistent across cognitive impairment levels (Supplement Table S.5 and S.6). Cognitive impairment was thus included as a covariate in the 3-subgroup LCA model to examine its association with physical frailty subgroups, with the “mild physical frailty” subgroup as the reference, adjusting for demographic and clinical characteristics (Table 4).

**Table 4:** Association between physical frailty subgroups and cognitive impairment in newly-admitted older nursing home residents^a^

	Moderate physical frailty subgroup *(*vs. *Mild physical frailty subgroup)*		Severe physical frailty subgroup *(*vs. *Mild physical frailty subgroup)*	
	aOR	95%CI	aOR	95%CI
**Cognitive impairment***(ref: none/mild)*				
Moderate	1.01	(0.99–1.03)	2.41	(2.35–2.47)
Severe	1.03	(1.01–1.05)	5.74	(5.58–5.90)
**Age***(ref: 65 - < 75 years)*				
75 - < 85 years	1.55	(1.52–1.58)	1.51	(1.47–1.54)
85 and over years	2.53	(2.48–2.59)	2.45	(2.38–2.51)
**Female***(ref: male)*	1.35	(1.32–1.37)	1.10	(1.08–1.13)
**Racial/ethnic minority***(ref: non-Hispanic white)*	0.76	(0.74–0.78)	1.84	(1.80–1.89)
**Rural nursing homes***(ref: urban)*	0.66	(0.65–0.67)	0.34	(0.33–0.35)
**Admission source***(ref: community)*				
Acute hospital	4.39	(4.31–4.48)	23.46	(23.62–25.33)
Other^b^	1.34	(1.31–1.37)	6.82	(6.58–7.06)
**Active diagnosis (***ref: without the diagnosis)*				
Cancer	1.11	(1.07–1.14)	1.12	(1.08–1.16)
Heart failure	1.46	(1.43–1.50)	1.35	(1.31–1.38)
Hypertension	1.06	(1.04–1.08)	0.96	(0.94–0.98)
Diabetes Mellitus	1.30	(1.28–1.33)	1.28	(1.26–1.31)
Cerebrovascular Accident/Transient Ischemic Attack/Stroke	1.47	(1.42–1.51)	4.97	(4.81–5.13)
Multiple Sclerosis	6.60	(5.41–8.05)	12.45	(10.09–15.36)
Parkinson’s Disease	2.57	(2.46–2.68)	4.87	(4.65–5.10)
Seizure disorder or Epilepsy	1.12	(1.08–1.16)	2.02	(1.94–2.11)
Arthritis	1.26	(1.24–1.28)	0.95	(0.93–0.99)
Osteoporosis	1.06	(1.04–1.09)	0.97	(0.93–1.00)
Hip fracture	8.20	(7.29–9.22)	13.35	(11.87–15.01)
Other fracture	3.87	(3.67–4.09)	3.24	(3.06–3.44)
Asthma/Chronic Obstructive Pulmonary Disease/Chronic Lung Disease	0.96	(0.94–0.97)	1.00	(0.98–1.02)
Anxiety disorder	0.91	(0.89–0.93)	0.87	(0.84–0.89)
Depression	1.04	(1.02–1.07)	1.04	(1.01–1.07)
**Any presence of pain***(ref: no presence)*	1.74	(1.71–1.77)	1.57	(1.54–1.60)
**Psychotropic medications received in past 7 days or since admission***(ref: did not receive)*				
Antipsychotics	0.64	(0.63–0.66)	0.61	(0.60–0.62)
Antianxiety	1.05	(1.02–1.07)	1.20	(1.17–1.23)
Antidepressant	1.14	(1.12–1.16)	1.10	(1.07–1.12)

^a^Multinomial logistic regression model adjusted for all demographic and clinical characteristics listed in this table

^b^Other admission sources included another nursing home or swing bed, psychiatric hospital, inpatient rehabilitation facility, ID/DD facility, long-term care hospitals, hospice, and other sources

Compared to those with none/mild cognitive impairment, older residents with moderate impairment had similar odds to belong to the “moderate physical frailty” subgroup (aOR: 1.01, 95%: 0.99–1.03), while over twice as likely (aOR: 2.41, 95%CI: 2.35–2.47) to belong to the “severe physical frailty” subgroup; older residents with severe impairment had slightly higher odds to belong to the “moderate physical frailty” subgroup (aOR: 1.03, 95%CI: 1.01–1.05), and were close to 6 times as likely (aOR: 5.74; 95%CI: 5.58–5.90) to belong to the “severe physical frailty” subgroup.

For demographic and clinical characteristics, older age and being female were associated with higher odds of belonging to the “moderate physical frailty” or “severe physical frailty” subgroups, compared to their respective counterparts. Older residents who were racial/ethnic minorities were less likely to belong to the “moderate physical frailty” subgroup, but more likely to belong to the “severe physical frailty” subgroup. Older residents in rural nursing homes were less likely to be in the “moderate physical frailty” or “severe physical frailty” subgroups than those in urban nursing homes. Older residents admitted from acute hospitals had much higher probabilities of belonging to the “moderate physical frailty” and “severe physical frailty” subgroup than those admitted from the community.

Older residents with cancer, heart failure, diabetes mellitus, cerebrovascular accident/TIA/stroke, multiple sclerosis, Parkinson’s disease, seizure disorder/epilepsy, hip fracture, other facture, or depression had higher odds of belong to the “moderate physical frailty” or “severe physical frailty” subgroups, while those with anxiety disorder had lower odds. Older residents with hypertension, arthritis or osteoporosis were more likely to belong to the “moderate physical frailty” subgroup, but less likely to be in the “severe physical frailty” subgroup. Older residents with any pain presence at admission were more likely to be in the “moderate physical frailty” or “severe physical frailty” subgroups. Older residents who received antipsychotics were less likely to be in the “moderate physical frailty” or “severe physical frailty” subgroups, while those who received antianxiety medications or antidepressants were more likely to do so.

Findings from sensitivity analysis suggested consistent positive association between cognitive impairment and physical frailty subgroups, but the magnitude of these associations increased (Supplement Table S.7). Particularly, in the absence of Alzheimer’s disease and non-Alzheimer’s/other dementia, older residents with severe cognitive impairment were 8.55 times (95% CI: 8.18–8.92) as likely to be in the “severe physical frailty” subgroup, compared to those with none/mild cognition.

## Discussion

In older adults in U.S. nursing homes, we identified three subgroups of physical frailty at nursing home admission, namely, “mild physical frailty”, “moderate physical frailty” and “severe physical frailty”. Physical frailty subgroups did not appear to differ across cognitive impairment levels. Older residents with greater levels of cognitive impairment were more likely to belong to the “moderate physical frailty” or “severe physical frailty” subgroups. Recent research has shown the possibility to reduce the prevalence or even reverse the progress of physical frailty through physical activity programs, cognitive training, nutritional supplementation, and interventions individualized to older adults’ clinical conditions [12, 13, 42]. However, these studies were conducted in community-dwelling older adults. Whether physical frailty could also serve as an intervention target for older nursing home residents warrants further exploration. To the best of our knowledge, this is the first study to provide evidence for the heterogeneity of physical frailty in older nursing home residents and its association with cognitive impairment, which can inform the development of interventions tailored to specific clinical profiles of physical frailty and cognitive impairment, while also considering the potential impact from other demographic and clinical characteristics.

The majority of the older nursing home residents in this study had high probabilities of belonging to either “moderate physical frailty” or “severe physical frailty” subgroups. This was expected as nearly two-thirds of the older nursing home residents were admitted post-hospitalization, indicating a more clinically complex group with greater care needs. The use of LCA allowed us to examine the heterogeneity of physical frailty by identifying three distinct subgroups. Regardless of the subgroups they were more likely to belong to, all residents had a high probability of requiring assistance with dressing. Besides the high probabilities of limited mobility that older residents belonging to the “moderate physical frailty” subgroup or the “severe physical frailty” subgroup were shown to have, those in the “severe physical frailty” subgroup also had particularly greater probability of bowel incontinence. Such distinctive experiences would be masked when physical frailty is measured by categorizing a total score into robust/pre-frail/frail levels. Using the LCA person-centered approach, findings not only reflected the increasing levels of physical frailty severity, but also provided a more nuanced picture of the physical frailty experience in older nursing home residents.

We note one important caveat that the characteristics of the subgroups to be identified by LCA is determined by the observed indicators, namely, FRAIL-NH items in the context of this study. Unique experiences of physical frailty in older nursing home residents that were not captured by FRAIL-NH would not be reflected in the identified subgroups. Therefore, other distinct subgroups of physical frailty may exist in older nursing home residents and future studies should consider additional metrics to provide a more comprehensive picture of the heterogeneity of physical frailty in this population.

The finding that greater levels of cognitive impairment was associated with increasingly higher odds to be in the “moderate physical frailty” and “severe physical frailty” subgroups provides additional evidence on the frequent co-occurrence of physical frailty and cognitive impairment, which has been established in older adults in the community [43, 44], but not in nursing homes. Further, in the sensitivity analysis when older residents with Alzheimer’s disease and those with non-Alzheimer’s/other dementia were excluded, the magnitude of the association between cognitive impairment and the “severe physical frailty” subgroup substantially increased, which could be indicative of “cognitive frailty”.

Regardless of older residents’ cognitive impairment levels, the characteristics of the identified physical frailty subgroups appeared to be similar, without notable differences in the patterns of the item-response probabilities*.* The consistent patterns of physical frailty subgroups were also observed in sensitivity analysis. These findings should be interpreted in light of the potential limitation of the instruments used to measure physical frailty and cognitive impairment. Despite several validation studies [4, 27–32], FRAIL-NH is admittedly a relatively new scale. Additionally, BIMS/CPS may not be informative for certain cognitive domains, such as executive functioning [35]. To further our understanding of the underlying mechanism between these two conditions in older adults in nursing homes, additional instruments that could provide a more granular, domain-specific measurement of both conditions are warranted.

Several demographic and clinical variables were also found to be associated with physical frailty subgroups, which may be helpful for care planning and triaging intervention efforts upon nursing home admission. Older age, being female and entering nursing homes from acute hospitals were associated with greater odds of belonging to the “moderate physical frailty” or “severe physical frailty” subgroups. It was unexpected that racial/ethnic minority older adults had lower odds of belonging to the “moderate physical frailty” subgroup and higher odds of belonging to the “severe physical frailty” subgroup. Future studies should attempt to elucidate and properly address the causes for the observed racial differences.

Consistent with prior studies that found pain [45], cancer [46], heart failure [47], diabetes [48], and depression [49] as risk factors for physical frailty in community-dwelling older adults, we provided additional information that older nursing home residents with these conditions would be more likely to belong to the “moderate physical frailty” or “severe physical frailty” subgroups. Although prior studies have demonstrated a strong positive relationship between frailty and Alzheimer’s and vascular dementia [50, 51], we did not include Alzheimer’s disease or non-Alzheimer’s/other dementia in the final model, as our preliminary findings suggested that a considerable extent of the impact on physical frailty subgroups from either of these two diagnoses would be through cognitive impairment.

Older adults who receive antipsychotics were less likely to be in the “moderate physical frailty” and “severe physical frailty” subgroups. Antipsychotics could be less prescribed to older residents in these two subgroups because they were more physically impaired, and thus less likely to have challenging behaviors that may have been handled using chemical restraints. The concerns that use of antipsychotics may increase risks for hospitalization and mortality in older adults who were frail [52, 53] may also play a role. Conversely, older residents who are more active and less frail may be more likely to receive antipsychotics because have a greater propensity to present behavioral management issues. Receipt of antidepressants was associated with higher odds of being in the “moderate physical frailty” or “severe physical frailty” subgroups. This may be attributed to the higher risks of functional limitations associated with antidepressant use [54]. On the other hand, the overlapping characteristics between depression and physical frailty may lead to an erroneous diagnosis of depression in those who were physically frail and not depressed, resulting in a wrong indication for antidepressant [55]. Given that MDS 3.0 only documenting the receipt of psychotropic medications in the past 7 days or since nursing home admission and the cross-sectional nature of the current study, we could not ascertain the clinical indications for these prescriptions and the length of time that the older adults have been using them, nor could we establish a causal relationship between psychotropic medications and physical frailty subgroups, explicitly, whether it was the concerns for physical frailty that influenced the prescription of these medications, or the use of these medications lead to a higher probability to belong to a certain physical frailty subgroup. However, considering that physical frailty may increase older adults’ vulnerability to adverse drug effects [52], additional research to examine their long-term impact on physical frailty could further inform the consideration of psychotropic medications in managing physical frailty in this population.

Limitations should be noted. Our analysis focused on older residents who stayed for longer than 100 days in nursing homes with life expectancy at admission longer than 6 months. If residents’ length of stay and/or life expectancy were differential with regards to symptoms of physical frailty, cognitive impairment levels, or other demographic and clinical characteristics, selection bias cannot be ruled out. This was a cross-sectional study at nursing home admission. As physical frailty and cognitive impairment could change during residents’ stay, longitudinal studies may be informative in exploring if and how physical frailty subgroups and cognitive impairment change over time.

## Conclusions

In summary, three subgroups of physical frailty were identified in older U.S. nursing home residents at admission, and older residents with greater levels of cognitive impairment were increasingly more likely to belong to the “moderate physical frailty” and “severe physical frailty” subgroups. Findings have implications for future efforts to tailor interventions to specific symptom profiles of physical frailty and cognitive impairment and provide new evidence for the interrelationship between these two prominent conditions in older nursing home residents.

## Supplementary Information

**Additional file 1.**

## Data Availability

Restrictions apply to the availability of the data (Minimum Data Set 3.0) under a data use agreement for this study. Minimum Data Set 3.0 is available from www.resdac.org with the permission of the Centers for Medicare and Medicaid Services.

## Abbreviations

IANA: International Academy on Nutrition and Aging
IAGG: International Association of Gerontology and Geriatrics
LCA: Latent class analysis
MDS 3.0: Minimum Data Set 3.0
BIMS: Brief Interview for Mental Status
CPS: Cognitive Performance Scale
ID/DD facility: Intellectual disabilities and developmental disabilities facility
TIA: Transient ischemic attack
COPD: Chronic obstructive pulmonary disease
AIC: Akaike Information Criterion
BIC: Bayesian Information Criterion
PHQ-9: Patient Health Questionnaire-9
