# Supplementary material for: Physical frailty and cognitive impairment in older nursing home residents: a latent class analysis
Source: BMC Geriatr. 2021 Sep 7;21:487. doi: 10.1186/s12877-021-02433-1 (PMC8425049; doi:10.1186/s12877-021-02433-1)
Supplement: Supplementary file 1 — Additional file 1. [file 12877_2021_2433_MOESM1_ESM.docx]

**Supplement File**

**Title:** Physical frailty and cognitive impairment in older nursing home residents: a latent class analysis

**Authors:**

Yiyang Yuan, MPH, MS, PhD Candidate ^1, 2^

Kate L. Lapane PhD, MS ^2^

Jennifer Tjia, MD, MSCE ^2^

Jonggyu Baek, PhD, MS ^2^

Shao-Hsien Liu, PhD, MPH ^2^

Christine M. Ulbricht, PhD, MPH ^3^

**Affiliations:**

**^1^** Clinical and Population Health Research PhD Program, Graduate School of Biomedical Sciences, University of Massachusetts Medical School, Worcester, MA, USA.

^2^ Department of Population and Quantitative Health Sciences, University of Massachusetts Medical School, Worcester, MA, USA.

^3^ National Institute of Mental Health, National Institutes of Health

**Corresponding author:** Yiyang Yuan, Department of Population and Quantitative Health Sciences, University of Massachusetts Medical School, 368 Plantation Street, Worcester, MA 01605. E-mail: [yiyang.yuan@umassmed.edu](mailto:yiyang.yuan@umassmed.edu).

**Supplement Method**

We examined if and how the subgroups of physical frailty would differ by cognitive impairment levels following these steps:

First, residents were stratified into subsets by cognitive impairment level at admission. Basic latent class analysis (LCA) models with 2-6 subgroups were fitted separately within each subset to evaluate if the number of physical frailty subgroups was identical across cognitive impairment levels. After considering the fit statistics, latent class prevalence and patterns of response probabilities, the classes appeared to be similar across all three cognitive impairment levels but (Supplement Table S.3) cognitive impairment was included as a grouping variable in the multiple-group LCA for the entire sample to test the measurement invariance assumption.

Second, we included cognitive impairment as a grouping variable in a multiple-group LCA model to determine whether residents with different cognitive impairment levels have the same physical frailty subgroups as represented by consistent patterns of item-response probabilities. Two sets of multiple-group models, one with (nested models) and the other without (full models) measurement invariance imposed, were fit and compared using the log-likelihood ratio test. Results from the log-likelihood difference test (Supplement Table S.4) showed a statistically significant difference between full models (without measurement invariance in item-response probabilities) and nested models (with measurement invariance), which suggested that the full model would better fit the data. As test statistics tend to be significant with large sample size, we also examined if the item-response probabilities of each subgroup were consistent between the full and nested models. It appeared that the patterns of item-response probabilities were consistent between full and nested models, supporting the measurement invariance assumption.

In conclusion, there appeared to be three physical frailty subgroups (“severe physical frailty”, “moderate physical frailty”, and “mild physical frailty”) in older nursing home residents, regardless of their level of cognitive impairment. Cognitive impairment was therefore included as a covariate in the logistic model to examine its association with the subgroups of physical frailty, adjusting for demographic and clinical characteristics.

**Supplement Tables and Figures**

**Supplement Figure S.1. Sample flowchart**

**Supplement Table S.1. The FRAIL-NH scale for physical frailty**

**Supplement Table S.2. Fit statistics of basic LCA models of physical frailty subgroups**

**Supplement Table S.3. Fit statistics of LCA models of physical frailty subgroups (Sensitivity Analysis)**

**Supplement Table S.4. Physical frailty 3-class latent class model: subgroup prevalence and item-response probabilities of indicators (Sensitivity Analysis)**

**Supplement Table S.5. Fit statistics of LCA models of physical frailty subgroups by cognitive impairment levels**

**Supplement Table S.6. Fit statistics of LCA models of physical frailty subgroups with cognitive impairment as a grouping variable and test of measurement invariance**

**Supplement Table S.7. Association between physical frailty subgroups and cognitive impairment in newly-admitted older nursing home residents (Sensitivity Analysis)**

**Supplement Figure S.1. Sample flowchart**

**Nursing home residents aged ≥ 65 years,**

**newly-admitted between January 1, 2014 and December 31, 2016**

n = 7,056,499

**Length of stay > 100 days (long-stay)**

n = 936,164

**Had prognosis of life expectancy ≥ 6 months at admission**

n = 875,604

**First episode was selected if a resident had multiple eligible nursing home episodes**

n = 872,659

**Not comatose at admission**

n = 871,801

**Note.** “Newly-admitted” was defined as having no nursing home episodes during a 90-day look-back period before the given nursing home admission. Life-expectancy was determined by a positive answer to the question “Does the resident have a condition or chronic disease that may result in a life expectancy of less than 6 months? (yes/no) on residents’ MDS 3.0 admission assessment.

**Supplement Table S.1. The FRAIL-NH scale for physical frailty ^a^**

| **Items** | **Item Score** | | | **Items in MDS 3.0** |
| --- | --- | --- | --- | --- |
|  | **0** | **1** | **2** |  |
| Fatigue ^b^ | No (never or 1 day) | Yes (several days or everyday) | PHQ-9 >= 10 | Section D Mood:  D0300/D0600; D0200-D/D0500-D |
| Resistance ^c^ | Independent | With set-up only | Need physical assistance | Section G Functional Status:  G0110B2 |
| Ambulation ^d^ | Independent | With assistive device (walker/cane) | Cannot walk | Section G Functional Status:  G0110E1; G0110F1; G0600A; G0600B; G0600C |
| Incontinence | None | Urinary incontinence only | Bowel incontinence | Section H Bladder and Bowel:  H0300; H0400 |
| Loss of weight | None | >= 5% in the past 3 months or >= 10% in the past 6 months | n/a ^e^ | Section K Swallowing/Nutritional Status:  K0300 |
| Nutritional approach | Regular diet | Mechanically altered diet | Require feeding tube | Section K Swallowing/Nutritional Status:  K0500 |
| Help with dressing | Independent | Need help with set up only | Need physical help | Section G Functional Status:  G0110G2 |

Note:

^a^ Kaehr EW, Pape LC, Malmstrom TK, Morley JE. FRAIL-NH predicts outcomes in long term care. J Nutr Health Aging. 2016;20(2):192-198. doi:10.1007/s12603-016-0682-5

^b^ Based on residents’ response to the Patient Health Questionnaire (PHQ-9; MDS 3.0 Section D: Mood).

^c^ Measures if the resident needs assistance to be transferred from one location to another.

^d^ Measures if the resident can walk in a room.

^e^ This item will only receive a score of 0 or 1. A score of 2 is not applicable.

**Supplement Table S.2. Fit statistics of basic LCA models of physical frailty subgroups**

| **Class** | **Entropy** | **AIC** | **BIC** | **adjusted BIC** | **Latent class prevalence** |
| --- | --- | --- | --- | --- | --- |
| 2 | 0.900 | 6113225.3 | 6113540.6 | 6113454.8 | 10.6%  89.4% |
| 3 | 0.621 | 6041188.5 | 6041667.3 | 6041537.0 | 7.6%  44.5%  47.9% |
| 4 | 0.664 | 6016312.5 | 6016954.9 | 6016780.1 | 3.7%  5.6%  43.6%  47.1% |
| 5 | 0.591 | 6012530.3 | 6013336.1 | 6013116.8 | 3.1%  3.6%  13.3%  38.5%  41.5% |
| 6 | 0.617 | 6010409.1 | 6011378.4 | 6011114.6 | 2.4%  2.9%  3.3%  11.8%  31.5%  48.1% |

**Supplement Table S.3. Fit statistics of LCA models of physical frailty subgroups (Sensitivity Analysis)**

*(A) Excluding older residents with diagnosis of Alzheimer’s disease (n=767,034)*

| **Class** | **Entropy** | **AIC** | **BIC** | **adjusted BIC** | **Latent class prevalence** |
| --- | --- | --- | --- | --- | --- |
| 2 | 0.906 | 5370229.657 | 5370541.514 | 5370455.707 | 10.0%  90.0% |
| 3 | 0.625 | 5306746.331 | 5307219.893 | 5307089.593 | 7.2%  47.7%  45.0% |
| 4 | 0.669 | 5284870.135 | 5285505.401 | 5285330.608 | 43.8%  5.4%  47.3%  3.5% |
| 5 | 0.591 | 5281820.349 | 5282617.318 | 5282398.033 | 3.0%  3.3%  12.8%  39.6%  41.3% |
| 6 | 0.640 | 5280238.572 | 5281197.246 | 5280933.468 | 3.3%  52.4%  28.7%  2.4%  2.8%  10.3% |

*(B) Excluding older residents with diagnosis of non- Alzheimer’s/other dementia (n=529,832)*

| **Class** | **Entropy** | **AIC** | **BIC** | **adjusted BIC** | **Latent class prevalence** |
| --- | --- | --- | --- | --- | --- |
| 2 | 0.914 | 3723735.482 | 3724037.350 | 3723951.543 | 90.6%  9.4% |
| 3 | 0.628 | 3680137.863 | 3680596.256 | 3680465.956 | 46.1%  7.0%  46.9% |
| 4 | 0.674 | 3664860.141 | 3665475.059 | 3665300.266 | 42.2%  5.1%  3.5%  49.2% |
| 5 | 0.602 | 3662777.881 | 3663549.322 | 3663330.037 | 2.9%  41.8%  11.8%  3.3%  40.1% |
| 6 | 0.642 | 3661592.379 | 3662520.345 | 3662256.567 | 10.5%  3.0%  27.7%  2.9%  53.5%  2.4% |

*(C) Excluding older residents with diagnosis of Alzheimer’s disease and those with non- Alzheimer’s/other dementia (n=460,612)*

| **Class** | **Entropy** | **AIC** | **BIC** | **adjusted BIC** | **Latent class prevalence** |
| --- | --- | --- | --- | --- | --- |
| 2 | 0.924 | 3222967.344 | 3223265.432 | 3223179.625 | 91.5%  8.5% |
| 3 | 0.641 | 3184427.659 | 3184880.312 | 3184750.012 | 6.4%  47.4%  46.2% |
| 4 | 0.684 | 3171050.680 | 3171657.897 | 3171483.104 | 42.9%  4.7%  49.2%  3.1% |
| 5 | 0.655 | 3169277.351 | 3170039.132 | 3169819.847 | 3.1%  12.5%  3.1%  24.5%  56.8% |
| 6 | 0.531 | 3168377.070 | 3169293.416 | 3169029.638 | 3.1%  24.4%  3.0%  12.1%  13.3%  44.1% |

**Supplement Table S.4. Physical frailty 3-class latent class model: subgroup prevalence and item-response probabilities of indicators (Sensitivity Analysis)**

*(A) Excluding older residents with diagnosis of Alzheimer’s disease (n=767,034)*

|  | | | **Mild physical frailty subgroup** | **Moderate physical frailty subgroup** | **Severe physical frailty subgroup** |
| --- | --- | --- | --- | --- | --- |
| **Subgroup prevalence** | | | 7.2% | 45.0% | 47.7% |
| **Item-response probabilities** | | |  |  |  |
| Fatigue | | |  |  |  |
|  | 0 | No (never or 1 day) | **0.72 *** | **0.60 *** | **0.61 *** |
|  | 1 | Yes (several days/everyday) | 0.23 | 0.35 | 0.32 |
|  | 2 | PHQ-9 ≥ 10 | 0.05 | 0.06 | 0.07 |
| Resistance ^1^ | | |  |  |  |
|  | 0 | Independent | **0.55 *** | 0.00 | 0.01 |
|  | 1 | With set-up only | 0.34 | 0.03 | 0.00 |
|  | 2 | Need physical assistance | 0.12 | **0.97 *** | **1.00 *** |
| Ambulation ^2^ | | |  |  |  |
|  | 0 | Independent | **0.53 *** | 0.05 | 0.01 |
|  | 1 | With assistive device | 0.19 | 0.13 | 0.01 |
|  | 2 | Cannot walk | 0.28 | **0.83 *** | **0.98 *** |
| Incontinence | | |  |  |  |
|  | 0 | None | **0.68 *** | 0.31 | 0.03 |
|  | 1 | Urinary incontinence only | 0.22 | **0.39 *** | 0.08 |
|  | 2 | Bowel incontinence | 0.10 | 0.30 | **0.89 *** |
| Loss of weight | | |  |  |  |
|  | 0 | None | **0.98 *** | **0.98 *** | **0.96 *** |
|  | 1 | ≥ 5% past 3 mo./ ≥10% past 6 mo. | 0.02 | 0.02 | 0.04 |
| Nutritional approach | | |  |  |  |
|  | 0 | Regular diet | **0.89 *** | **0.84 *** | **0.49 *** |
|  | 1 | Mechanically altered diet | 0.10 | 0.15 | 0.40 |
|  | 2 | Require feeding tube | 0.01 | 0.01 | 0.11 |
| Help with dressing | | |  |  |  |
|  | 0 | Independent | 0.25 | 0.00 | 0.00 |
|  | 1 | Need help with set up only | 0.35 | 0.01 | 0.00 |
|  | 2 | Need physical help | **0.41 *** | **0.99 *** | **1.00 *** |

*(B) Excluding older residents with diagnosis of non- Alzheimer’s/other dementia (n=529,832)*

|  | | | **Mild physical frailty subgroup** | **Moderate physical frailty subgroup** | **Severe physical frailty subgroup** |
| --- | --- | --- | --- | --- | --- |
| **Subgroup prevalence** | | | 7.0% | 46.9% | 46.1% |
| **Item-response probabilities** | | |  |  |  |
| Fatigue | | |  |  |  |
|  | 0 | No (never or 1 day) | **0.71 *** | **0.58 *** | **0.60 *** |
|  | 1 | Yes (several days/everyday) | 0.24 | 0.36 | 0.33 |
|  | 2 | PHQ-9 ≥ 10 | 0.05 | 0.06 | 0.07 |
| Resistance ^1^ | | |  |  |  |
|  | 0 | Independent | **0.55 *** | 0.00 | 0.01 |
|  | 1 | With set-up only | 0.33 | 0.02 | 0.00 |
|  | 2 | Need physical assistance | 0.12 | **0.98 *** | **1.00 *** |
| Ambulation ^2^ | | |  |  |  |
|  | 0 | Independent | **0.55 *** | 0.05 | 0.01 |
|  | 1 | With assistive device | 0.19 | 0.11 | 0.01 |
|  | 2 | Cannot walk | 0.26 | **0.84 *** | **0.99 *** |
| Incontinence | | |  |  |  |
|  | 0 | None | **0.69 *** | 0.33 | 0.03 |
|  | 1 | Urinary incontinence only | 0.22 | **0.39 *** | 0.08 |
|  | 2 | Bowel incontinence | 0.10 | 0.2 | **0.89 *** |
| Loss of weight | | |  |  |  |
|  | 0 | None | **0.98 *** | **0.97 *** | **0.96 *** |
|  | 1 | ≥ 5% past 3 mo./ ≥10% past 6 mo. | 0.02 | 0.03 | 0.04 |
| Nutritional approach | | |  |  |  |
|  | 0 | Regular diet | **0.90 *** | **0.84 *** | **0.50 *** |
|  | 1 | Mechanically altered diet | 0.10 | 0.15 | 0.37 |
|  | 2 | Require feeding tube | 0.01 | 0.01 | 0.13 |
| Help with dressing | | |  |  |  |
|  | 0 | Independent | 0.27 | 0.00 | 0.00 |
|  | 1 | Need help with set up only | 0.34 | 0.01 | 0.00 |
|  | 2 | Need physical help | **0.39 *** | **0.99 *** | **1.00 *** |

*(C) Excluding older residents with diagnosis of Alzheimer’s disease and those with non- Alzheimer’s/other dementia (n=460,612)*

|  | | | **Mild physical frailty subgroup** | **Moderate physical frailty subgroup** | **Severe physical frailty subgroup** |
| --- | --- | --- | --- | --- | --- |
| **Subgroup prevalence** | | | 6.4% | 47.4% | 46.2% |
| **Item-response probabilities** | | |  |  |  |
| Fatigue | | |  |  |  |
|  | 0 | No (never or 1 day) | **0.69 *** | **0.57 *** | **0.59 *** |
|  | 1 | Yes (several days/everyday) | 0.26 | 0.37 | 0.34 |
|  | 2 | PHQ-9 ≥ 10 | 0.05 | 0.06 | 0.07 |
| Resistance ^1^ | | |  |  |  |
|  | 0 | Independent | **0.54 *** | 0.00 | 0.00 |
|  | 1 | With set-up only | 0.34 | 0.02 | 0.00 |
|  | 2 | Need physical assistance | 0.12 | **0.98 *** | **1.00 *** |
| Ambulation ^2^ | | |  |  |  |
|  | 0 | Independent | **0.55 *** | 0.05 | 0.01 |
|  | 1 | With assistive device | 0.20 | 0.10 | 0.01 |
|  | 2 | Cannot walk | 0.25 | **0.85 *** | **0.99 *** |
| Incontinence | | |  |  |  |
|  | 0 | None | **0.71 *** | 0.35 | 0.03 |
|  | 1 | Urinary incontinence only | 0.21 | **0.39 *** | 0.08 |
|  | 2 | Bowel incontinence | 0.08 | 0.26 | **0.90 *** |
| Loss of weight | | |  |  |  |
|  | 0 | None | **0.98 *** | **0.97 *** | **0.96 *** |
|  | 1 | ≥ 5% past 3 mo./ ≥10% past 6 mo. | 0.02 | 0.03 | 0.04 |
| Nutritional approach | | |  |  |  |
|  | 0 | Regular diet | **0.89 *** | **0.84 *** | **0.51 *** |
|  | 1 | Mechanically altered diet | 0.10 | 0.15 | 0.35 |
|  | 2 | Require feeding tube | 0.01 | 0.01 | 0.14 |
| Help with dressing | | |  |  |  |
|  | 0 | Independent | 0.29 | 0.00 | 0.00 |
|  | 1 | Need help with set up only | 0.34 | 0.01 | 0.00 |
|  | 2 | Need physical help | **0.36 *** | **0.99 *** | **1.00 *** |

Note. PHQ-9 = Patient Health Questionnaire-9.

* The level of the given indicator with the highest item-response probability. Residents belonging to the given subgroup had the highest probability of experiencing this level of the indicator.

^1^ Measures if the resident needs assistance to be transferred from one location to another.

^2^ Measures if the resident can walk in a room.

**Supplement Table S.5. Fit statistics of LCA models of physical frailty subgroups by cognitive impairment levels**

| **Class** | **Entropy** | **AIC** | **BIC** | **adjusted BIC** | **Latent class prevalence** |
| --- | --- | --- | --- | --- | --- |
| Cognitively intact (n = 292,548) | | | | | |
| 2 | 0.932 | 2080430.7 | 2080716.6 | 2080630.8 | 9.0%; 90.2% |
| 3 | 0.595 | 2065296.9 | 2065730.9 | 2065600.6 | 8.1%; 43.0%; 48.9% |
| 4 | 0.648 | 2054145.3 | 2054727.5 | 2054552.7 | 4.1%; 5.8%; 41.3%; 48.8% |
| 5 | 0.668 | 2052883.1 | 2053613.6 | 2053394.3 | 3.7%; 4.1%; 13.6%; 17.0%; 61.5% |
| 6 | 0.567 | 2052426.9 | 2053305.5 | 2053041.7 | 3.6%; 4.1%; 7.4%; 13.1%; 20.0%; 51.8% |
|  |  |  |  |  |  |
| Moderate cognitive impairment (n = 262,307) | | | | | |
| 2 | 0.904 | 1851917.2 | 1852200.0 | 1852114.2 | 10.7%; 89.3% |
| 3 | 0.618 | 1831981.8 | 1832411.4 | 1832281.1 | 7.8%; 41.2%; 51.0% |
| 4 | 0.660 | 1824164.7 | 1824740.9 | 1824566.2 | 3.7%; 5.9%; 43.6%; 46.7% |
| 5 | 0.591 | 1822984.5 | 1823707.4 | 1823488.1 | 3.2%; 3.7%; 13.0%; 35.4%; 44.7% |
| 6 | 0.708 | 1822382.8 | 1823252.4 | 1822988.6 | 2.2%; 3.0%; 3.8%; 11.6%; 19.8%; 59.7% |
|  |  |  |  |  |  |
| Severe cognitive impairment (n = 316,946) | | | | | |
| 2 | 0.849 | 2113054.8 | 2113342.7 | 2113256.9 | 12.8%; 87.2% |
| 3 | 0.635 | 2082990.3 | 2083427.6 | 2083297.3 | 7.2%; 40.1%; 52.7% |
| 4 | 0.672 | 2076901.8 | 2077488.4 | 2077313.6 | 3.5%; 5.1%; 42.4%; 48.9% |
| 5 | 0.682 | 2075411.1 | 2076147.1 | 2075927.8 | 2.4%; 4.2%; 4.4%; 39.6%; 49.4% |
| 6 | 0.582 | 2074387.6 | 2075273.0 | 2075009.2 | 2.3%; 3.1%; 3.2%; 15.4%; 38.0%; 38.0% |

**Supplement Table S.6. Fit statistics of LCA models of physical frailty subgroups with cognitive impairment as a grouping variable and test of measurement invariance**

| **LCA model** | **Entropy** | **AIC** | **BIC** | **adjusted BIC** | **Log-likelihood** | **Scaling correction factor for MLR** | **Number of free parameters** | **Log-likelihood ratio test p-value** |
| --- | --- | --- | --- | --- | --- | --- | --- | --- |
| 2-class without MI | 0.959 | 7955768.08 | 7956737.38 | 7956473.60 | -3977801.04 (a) | 1.0459 (c) | 83 (e) | <0.0001 |
| 2-class with MI | 0.960 | 8022526.91 | 8022888.94 | 8022790.42 | -4011232.46 (b) | 1.0395 (d) | 31 (f) |  |
|  |  |  |  |  |  |  |  |  |
| 3-class without MI | 0.808 | 7890634.37 | 7892094.16 | 7891696.90 | -3945192.18 | 1.0240 | 125 | <0.0001 |
| 3-class with MI | 0.827 | 7917163.80 | 7917712.68 | 7917563.32 | -3958534.90 | 1.0243 | 47 |  |
|  |  |  |  |  |  |  |  |  |
| 4-class without MI | 0.811 | 7865577.24 | 7867527.52 | 7866996.79 | -3932621.62 | 1.0135 | 167 | <0.0001 |
| 4-class with MI | 0.829 | 7888986.17 | 7889721.90 | 7889521.69 | -3944430.08 | 1.0252 | 63 |  |
|  |  |  |  |  |  |  |  |  |
| 5-class without MI | 0.767 | 7861856.79 | 7864297.56 | 7863633.34 | -3930719.39 | 1.0279 | 209 | <0.0001 |
| 5-class with MI | 0.799 | 7874810.16 | 7875732.75 | 7875481.68 | -3937326.08 | 1.0256 | 79 |  |
|  |  |  |  |  |  |  |  |  |
| 6-class without MI | 0.761 | 7859558.05 | 7862489.31 | 7861691.62 | -3929528.03 | 1.0449 | 251 | <0.0001 |
| 6-class with MI | 0.724 | 7869727.16 | 7870836.60 | 7870534.68 | -3934768.58 | 1.0388 | 95 |  |

Note:

MI: Measurement invariance across cognitive impairment levels

Log-likelihood ratio test p-value calculation: Cd = [(c*e)-(d*f)]/(e-f); LR = 2*(b-a)/Cd; Df = e-f; then use chi-square distribution to determine the p-value for LR with Df degrees of freedom.

**Supplement Table S.7. Association between physical frailty subgroups and cognitive impairment in newly-admitted older nursing home residents^1^ (Sensitivity Analysis)**

*(A) Excluding older residents with diagnosis of Alzheimer’s disease (n=767,034)*

|  |  | **Moderate physical frailty subgroup**  *(vs. Mild physical frailty subgroup)* | | **Severe physical frailty subgroup**  *(vs. Mild physical frailty subgroup)* | |
| --- | --- | --- | --- | --- | --- |
|  |  | aOR | 95%CI | aOR | 95%CI |
| **Cognitive impairment** *(ref: none/mild)* | |  |  |  |  |
|  | Moderate | 1.04 | (1.02-1.06) | 2.51 | (2.44-2.57) |
|  | Severe | 1.07 | (1.05-1.10) | 6.14 | (5.96-6.32) |
| **Age** *(ref: 65 - <75 years)* | |  |  |  |  |
|  | 75 - <85 years | 1.57 | (1.53-1.60) | 1.53 | (1.49-1.57) |
|  | 85 and over years | 2.48 | (2.42-2.53) | 2.33 | (2.26-2.40) |
| **Female** *(ref: male)* | | 1.37 | (1.35-1.40) | 1.12 | (1.10-1.15) |
| **Racial/ethnic minority** *(ref: non-Hispanic white)* | | 0.77 | (0.75-0.79) | 1.84 | (1.79-1.89) |
| **Rural nursing homes** *(ref: urban)* | | 0.65 | (0.64-0.66) | 0.32 | (0.32-0.33) |
| **Admission source** *(ref: community)* | |  |  |  |  |
|  | Acute hospital | 4.72 | (4.62-4.83) | 28.50 | (27.40-29.65) |
|  | Other ^2^ | 1.42 | (1.39-1.46) | 7.76 | (7.46-8.08) |
| **Active diagnosis (***ref: without the diagnosis)* | |  |  |  |  |
|  | Cancer | 1.11 | (1.07-1.14) | 1.13 | (1.08-1.17) |
|  | Heart failure | 1.45 | (1.41-1.48) | 1.31 | (1.27-1.34) |
|  | Hypertension | 1.06 | (1.04-1.08) | 0.98 | (0.95-1.00) |
|  | Diabetes Mellitus | 1.30 | (1.28-1.33) | 1.28 | (1.25-1.31) |
|  | Cerebrovascular Accident/Transient Ischemic Attack/Stroke | 1.49 | (1.44-1.54) | 5.24 | (5.07-5.43) |
|  | Multiple Sclerosis | 6.77 | (5.52-8.32) | 12.71 | (10.22-15.80) |
|  | Parkinson's Disease | 2.58 | (2.46-2.70) | 4.80 | (4.56-5.04) |
|  | Seizure disorder or Epilepsy | 1.11 | (1.06-1.16) | 1.99 | (1.90-2.07) |
|  | Arthritis | 1.26 | (1.23-1.28) | 0.92 | (0.90-0.95) |
|  | Osteoporosis | 1.06 | (1.03-1.09) | 0.93 | (0.90-0.96) |
|  | Hip fracture | 7.91 | (7.00-8.94) | 11.86 | (10.49-13.41) |
|  | Other fracture | 3.77 | (3.56-4.00) | 3.07 | (2.89-3.27) |
|  | Asthma/Chronic Obstructive Pulmonary Disease/Chronic Lung Disease | 0.94 | (0.92-0.96) | 0.99 | (0.96-1.01) |
|  | Anxiety disorder | 0.91 | (0.89-0.94) | 0.85 | (0.83-0.88) |
|  | Depression | 1.04 | (1.02-1.07) | 1.04 | (1.01-1.07) |
| **Any presence of pain** *(ref: no presence)* | | 1.74 | (1.71-1.78) | 1.57 | (1.53-1.60) |
| **Psychotropic medications received in past 7 days or since admission** *(ref: did not receive)* | |  |  |  |  |
|  | Antipsychotics | 0.60 | (0.58-0.61) | 0.57 | (0.56-0.59) |
|  | Antianxiety | 1.01 | (0.99-1.04) | 1.17 | (1.14-1.21) |
|  | Antidepressant | 1.16 | (1.13-1.19) | 1.13 | (1.10-1.16) |

*(B) Excluding older residents with diagnosis of non- Alzheimer’s/other dementia (n=529,832)*

|  |  | **Moderate physical frailty subgroup**  *(vs. Mild physical frailty subgroup)* | | **Severe physical frailty subgroup**  *(vs. Mild physical frailty subgroup)* | |
| --- | --- | --- | --- | --- | --- |
|  |  | aOR | 95%CI | aOR | 95%CI |
| **Cognitive impairment** *(ref: none/mild)* | |  |  |  |  |
|  | Moderate | 1.08 | (1.05-1.11) | 2.77 | (2.68-2.86) |
|  | Severe | 1.06 | (1.03-1.09) | 6.90 | (6.64-7.16) |
| **Age** *(ref: 65 - <75 years)* | |  |  |  |  |
|  | 75 - <85 years | 1.55 | (1.51-1.59) | 1.51 | (1.46-1.56) |
|  | 85 and over years | 2.29 | (2.23-2.36) | 1.94 | (1.87-2.01) |
| **Female** *(ref: male)* | | 1.39 | (1.36-1.42) | 1.15 | (1.12-1.18) |
| **Racial/ethnic minority** *(ref: non-Hispanic white)* | | 0.83 | (0.80-0.85) | 1.89 | (1.83-1.95) |
| **Rural nursing homes** *(ref: urban)* | | 0.63 | (0.62-0.65) | 0.32 | (0.31-0.33) |
| **Admission source** *(ref: community)* | |  |  |  |  |
|  | Acute hospital | 5.46 | (5.32-5.61) | 33.40 | (31.75-35.12) |
|  | Other ^2^ | 1.51 | (1.47-1.56) | 8.68 | (8.24-9.14) |
| **Active diagnosis (***ref: without the diagnosis)* | |  |  |  |  |
|  | Cancer | 1.09 | (1.04-1.13) | 1.14 | (1.08-1.19) |
|  | Heart failure | 1.41 | (1.37-1.45) | 1.19 | (1.15-1.23) |
|  | Hypertension | 1.07 | (1.05-1.10) | 0.98 | (0.95-1.00) |
|  | Diabetes Mellitus | 1.30 | (1.27-1.33) | 1.23 | (1.20-1.27) |
|  | Cerebrovascular Accident/Transient Ischemic Attack/Stroke | 1.56 | (1.50-1.63) | 6.19 | (5.92-6.46) |
|  | Multiple Sclerosis | 6.87 | (5.45-8.65) | 11.63 | (9.07-14.91) |
|  | Parkinson's Disease | 2.38 | (2.24-2.52) | 4.06 | (3.80-4.33) |
|  | Seizure disorder or Epilepsy | 1.16 | (1.10-1.22) | 2.04 | (1.93-2.15) |
|  | Arthritis | 1.25 | (1.22-1.28) | 0.88 | (0.86-0.91) |
|  | Osteoporosis | 1.05 | (1.02-1.09) | 0.90 | (0.87-0.95) |
|  | Hip fracture | 7.10 | (6.18-8.14) | 8.81 | (7.66-10.14) |
|  | Other fracture | 3.94 | (3.68-4.22) | 2.90 | (2.68-3.13) |
|  | Asthma/Chronic Obstructive Pulmonary Disease/Chronic Lung Disease | 0.90 | (0.88-0.93) | 0.94 | (0.91-0.97) |
|  | Anxiety disorder | 0.92 | (0.89-0.95) | 0.87 | (0.83-0.90) |
|  | Depression | 1.04 | (1.01-1.07) | 1.04 | (1.00-1.08) |
| **Any presence of pain** *(ref: no presence)* | | 1.74 | (1.70-1.78) | 1.53 | (1.49-1.57) |
| **Psychotropic medications received in past 7 days or since admission** *(ref: did not receive)* | |  |  |  |  |
|  | Antipsychotics | 0.55 | (0.54-0.57) | 0.52 | (0.50-0.54) |
|  | Antianxiety | 1.01 | (0.98-1.05) | 1.16 | (1.11-1.20) |
|  | Antidepressant | 1.13 | (1.10-1.16) | 1.09 | (1.05-1.13) |

*(C) Excluding older residents with diagnosis of Alzheimer’s disease and those with non- Alzheimer’s/other dementia (n=460,612)*

|  |  | **Moderate physical frailty subgroup**  *(vs. Mild physical frailty subgroup)* | | **Severe physical frailty subgroup**  *(vs. Mild physical frailty subgroup)* | |
| --- | --- | --- | --- | --- | --- |
|  |  | aOR | 95%CI | aOR | 95%CI |
| **Cognitive impairment** *(ref: none/mild)* | |  |  |  |  |
|  | Moderate | 1.17 | (1.14-1.20) | 3.00 | (2.90-3.10) |
|  | Severe | 1.22 | (1.17-1.26) | 8.55 | (8.18-8.92) |
| **Age** *(ref: 65 - <75 years)* | |  |  |  |  |
|  | 75 - <85 years | 1.60 | (1.55-1.64) | 1.58 | (1.52-1.63) |
|  | 85 and over years | 2.15 | (2.01-2.22) | 1.70 | (1.63-1.76) |
| **Female** *(ref: male)* | | 1.45 | (1.42-1.48) | 1.17 | (1.14-1.21) |
| **Racial/ethnic minority** *(ref: non-Hispanic white)* | | 0.84 | (0.82-0.87) | 1.87 | (1.81-1.94) |
| **Rural nursing homes** *(ref: urban)* | | 0.63 | (0.61-0.64) | 0.31 | (0.30-0.32) |
| **Admission source** *(ref: community)* | |  |  |  |  |
|  | Acute hospital | 5.70 | (5.54-5.86) | 39.75 | (37.41-42.24) |
|  | Other ^2^ | 1.65 | (1.60-1.71) | 11.01 | (10.34-11.73) |
| **Active diagnosis (***ref: without the diagnosis)* | |  |  |  |  |
|  | Cancer | 1.05 | (1.01-1.09) | 1.12 | (1.07-1.18) |
|  | Heart failure | 1.35 | (1.31-1.39) | 1.09 | (1.05-1.13) |
|  | Hypertension | 1.08 | (1.05-1.11) | 1.00 | (0.97-1.04) |
|  | Diabetes Mellitus | 1.27 | (1.24-1.30) | 1.18 | (1.14-1.21) |
|  | Cerebrovascular Accident/Transient Ischemic Attack/Stroke | 1.53 | (1.46-1.59) | 6.37 | (6.08-6.66) |
|  | Multiple Sclerosis | 6.09 | (4.89-7.57) | 9.43 | (7.42-11.99) |
|  | Parkinson's Disease | 2.26 | (2.13-2.40) | 3.64 | (3.40-3.90) |
|  | Seizure disorder or Epilepsy | 1.13 | (1.07-1.19) | 1.95 | (1.84-2.07) |
|  | Arthritis | 1.25 | (1.22-1.28) | 0.84 | (0.81-0.87) |
|  | Osteoporosis | 1.03 | (1.00-1.07) | 0.85 | (0.81-0.89) |
|  | Hip fracture | 6.31 | (5.53-7.20) | 6.47 | (5.64-7.42) |
|  | Other fracture | 3.56 | (3.33-3.81) | 2.43 | (2.25-2.62) |
|  | Asthma/Chronic Obstructive Pulmonary Disease/Chronic Lung Disease | 0.87 | (0.84-0.89) | 0.91 | (0.88-0.94) |
|  | Anxiety disorder | 0.93 | (0.90-0.96) | 0.87 | (0.83-0.91) |
|  | Depression | 1.04 | (1.01-1.08) | 1.04 | (1.00-1.08) |
| **Any presence of pain** *(ref: no presence)* | | 1.71 | (1.67-1.75) | 1.46 | (1.42-1.50) |
| **Psychotropic medications received in past 7 days or since admission** *(ref: did not receive)* | |  |  |  |  |
|  | Antipsychotics | 0.48 | (0.47-0.49) | 0.49 | (0.47-0.51) |
|  | Antianxiety | 0.95 | (0.92-0.99) | 1.11 | (1.07-1.16) |
|  | Antidepressant | 1.16 | (1.12-1.19) | 1.13 | (1.09-1.18) |

Note: TIA = transient ischemic attack; COPD = chronic obstructive pulmonary disease; aOR = adjusted odds ratio; 95%CI = 95% confidence interval.

^1^ Measured by FRAIL-NH using previously validated cutoffs: robust (0-5), pre-frail (6-7), and frail (≥8).

^2^ Included another nursing home/swing bed, psychiatric hospital, inpatient rehabilitation facility, intellectual disabilities and developmental disabilities (ID/DD) facility, long-term care hospitals, hospice, and other unspecified admission sources.
